# Supplementary material for: Inhibition of D-Ala:D-Ala ligase through a phosphorylated form of the antibiotic D-cycloserine
Source: Nat Commun. 2017 Dec 5;8:1939. doi: 10.1038/s41467-017-02118-7 (PMC5717164; doi:10.1038/s41467-017-02118-7)
Supplement: Supplementary file 1 — Supplementary Information [file 41467_2017_2118_MOESM1_ESM.pdf]

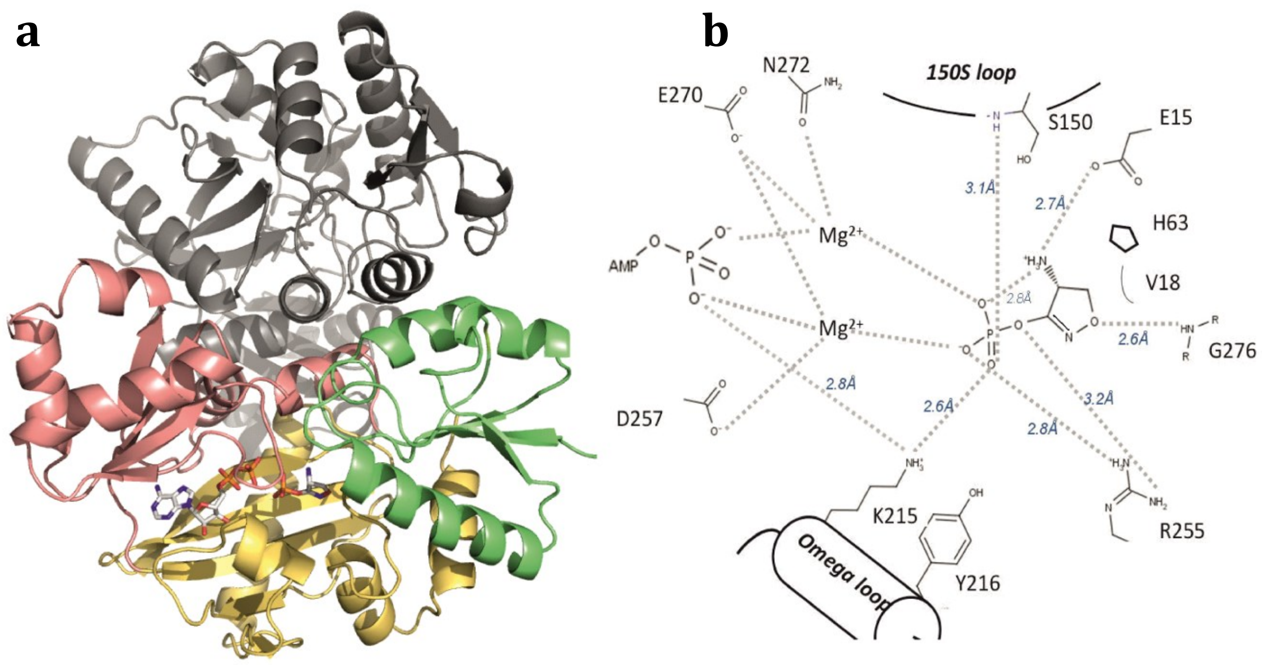

Supplementary Figure 1a. Cartoon representation of the overall structure of EcDdlB. Structure of EcDdlB in complex with ATP and DCSP. Dimer chain A is colored as described below, dimer chain B is shown in grey. The dimer structure of *E. coli* DdlB has been described previously [1]. It is divided into 3 domains, each with an α/β structure: an N-terminal domain (residues 1-85: green), a central domain (residues 86-180: salmon pink) and a C-terminal domain (residues 181-306: yellow). The nucleotide binding site is located between the central and C-terminal domains and is made up of three anti-parallel β-strands of the C-terminal domain, and the wall of the crevice is formed by three loops: loop I (residues 13-16), loop II (150s loop: residues 147-153) containing the active site Ser-150, and loop III (omega loop: residues 210-218) which covers both ATP and D-alanine ligand binding sites.

Supplementary Figure 1b. Ligplot representation of residues in contact with ADP and DCSP. Hydrogen bond distances are represented as dashed lines with distances in Å.

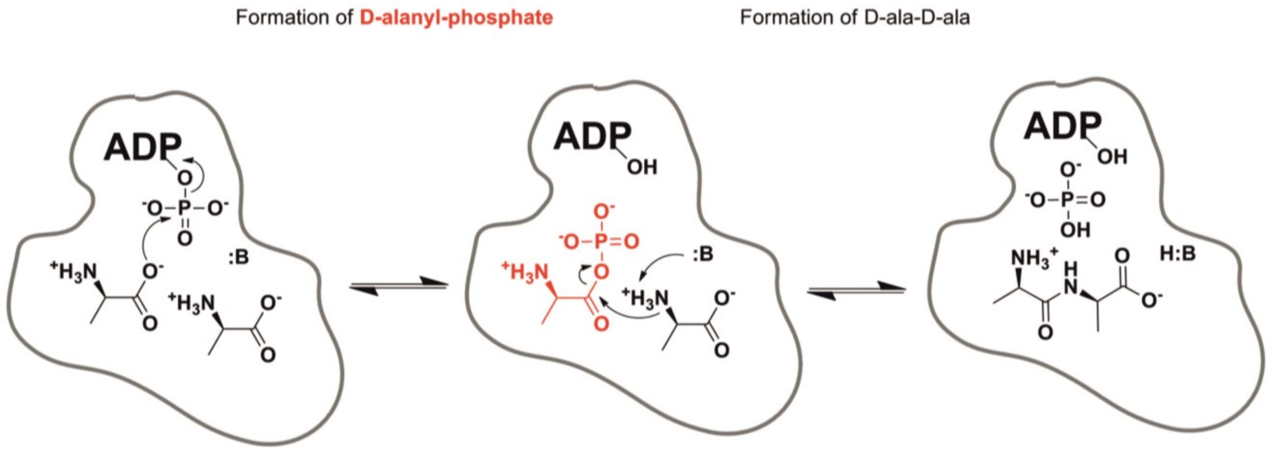

Supplementary Figure 2. Mechanism of D-Ala-D-Ala formation via D-alanyl-phosphate intermediate.

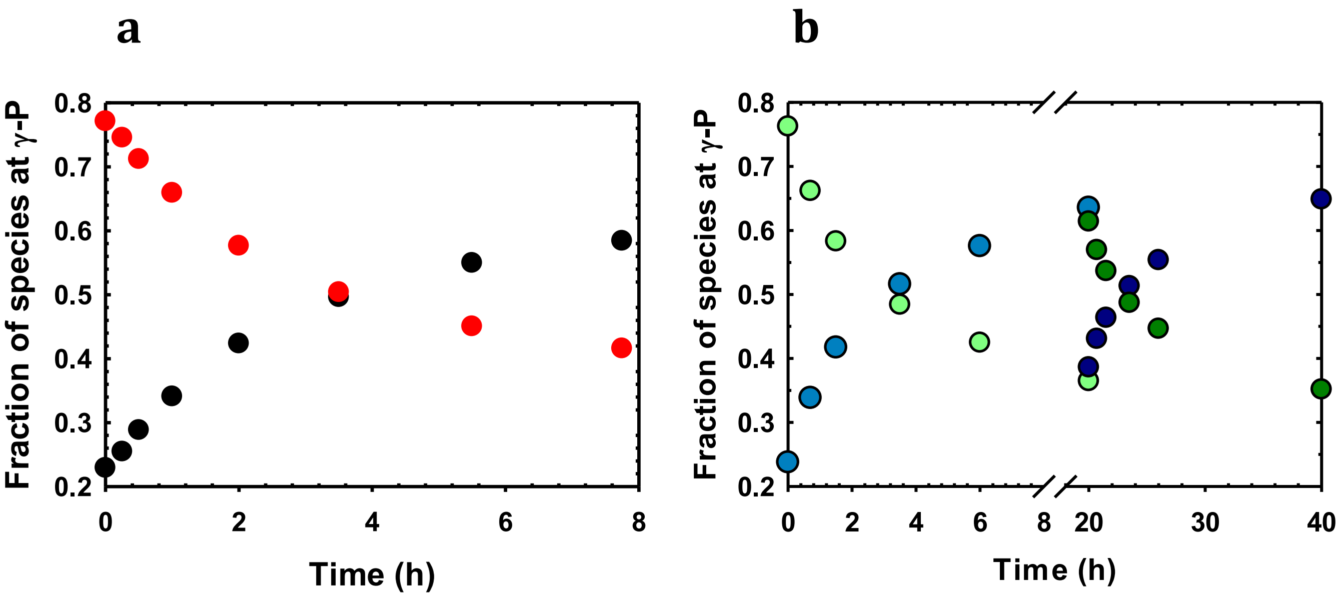

Supplementary Figure 3: Positional isotope exchange catalysed by EcDdlB in the presence of DCS and [γ-$^{18}O_4$]-ATP. Panel (a), change in the fraction of species at γ-P of [γ-$^{18}O_4$]-ATP during the PIX reaction at 25°C as monitored by ^{31}P- NMR, $^{18}O_3{}^{16}O$ (black) and $^{18}O_4$ (red). Panel (b), control experiment to probe the mechanistic origin of the decrease in exchange rate. Addition of fresh [γ-$^{18}O_4$]-ATP leads to identical exchange kinetics, indicating that the equilibrium reached is not caused by enzyme inactivation or denaturation, but a true isotopic equilibrium. PIX reaction after 20 hours: fraction of $^{18}O_3{}^{16}O$ (light blue) and $^{18}O_4$ (light green) species at γ-P monitored over 20 hours from the beginning of the reaction and for subsequent 20 hours after addition to the reaction mix of further 2 mM of [γ-$^{18}O_4$]-ATP, $^{18}O_3{}^{16}O$ (dark blue) and $^{18}O_4$ (dark green).

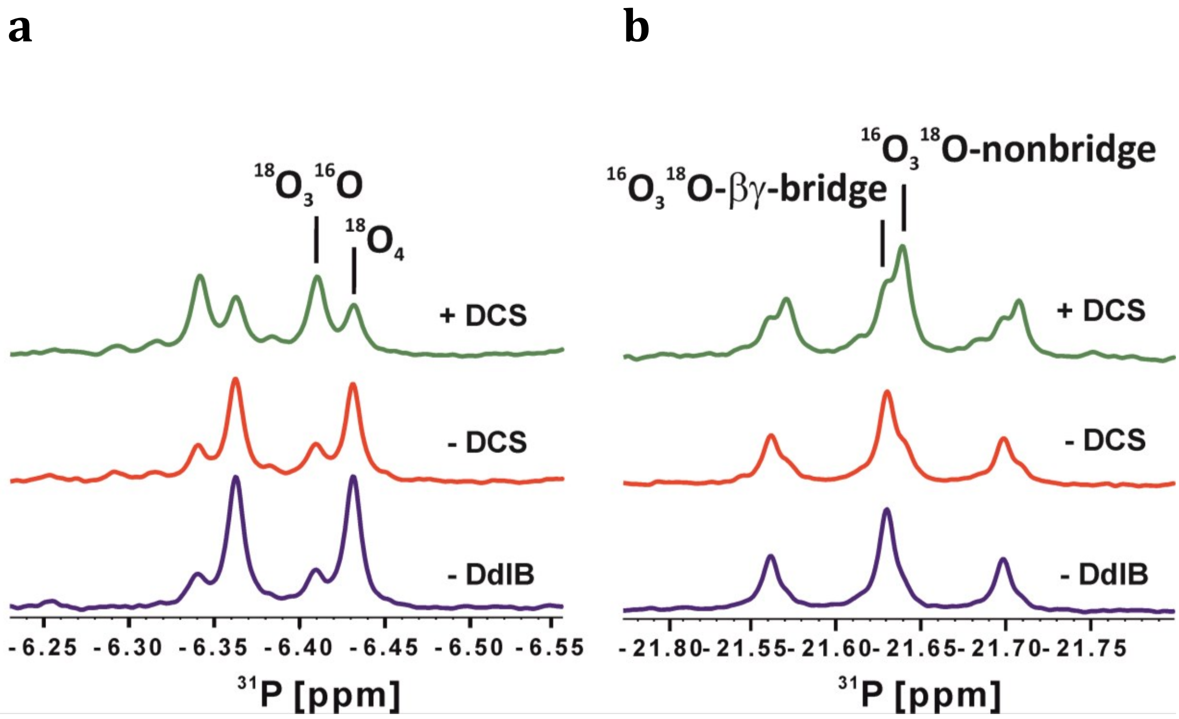

Supplementary Figure 4. PIX experiment controls at γ-P (a) and β-P (b) of [γ-$^{18}O_4$]-ATP. ^{31}P-NMR spectra after 12 h incubation at 25 °C in the presence (green) and absence (red) of DCS show that the reaction happens only in the presence of the DCS. Reference spectrum of a DCS containing sample not treated with EcDdlB (blue).

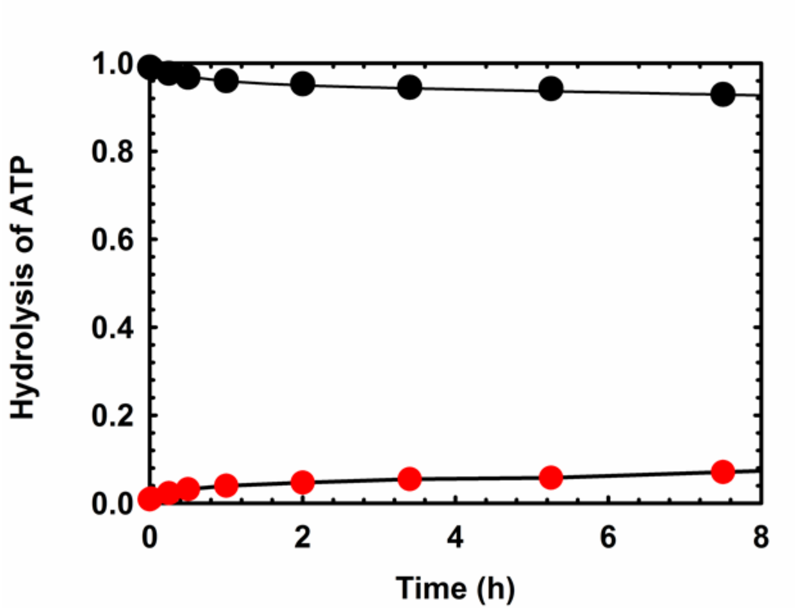

Supplementary Figure 5. Hydrolysis of ATP as monitored by ^{31}P-NMR under the PIX experiment conditions. Fractions of ATP (black) and ADP (red) species calculated by integration of the α-P doublet peak.

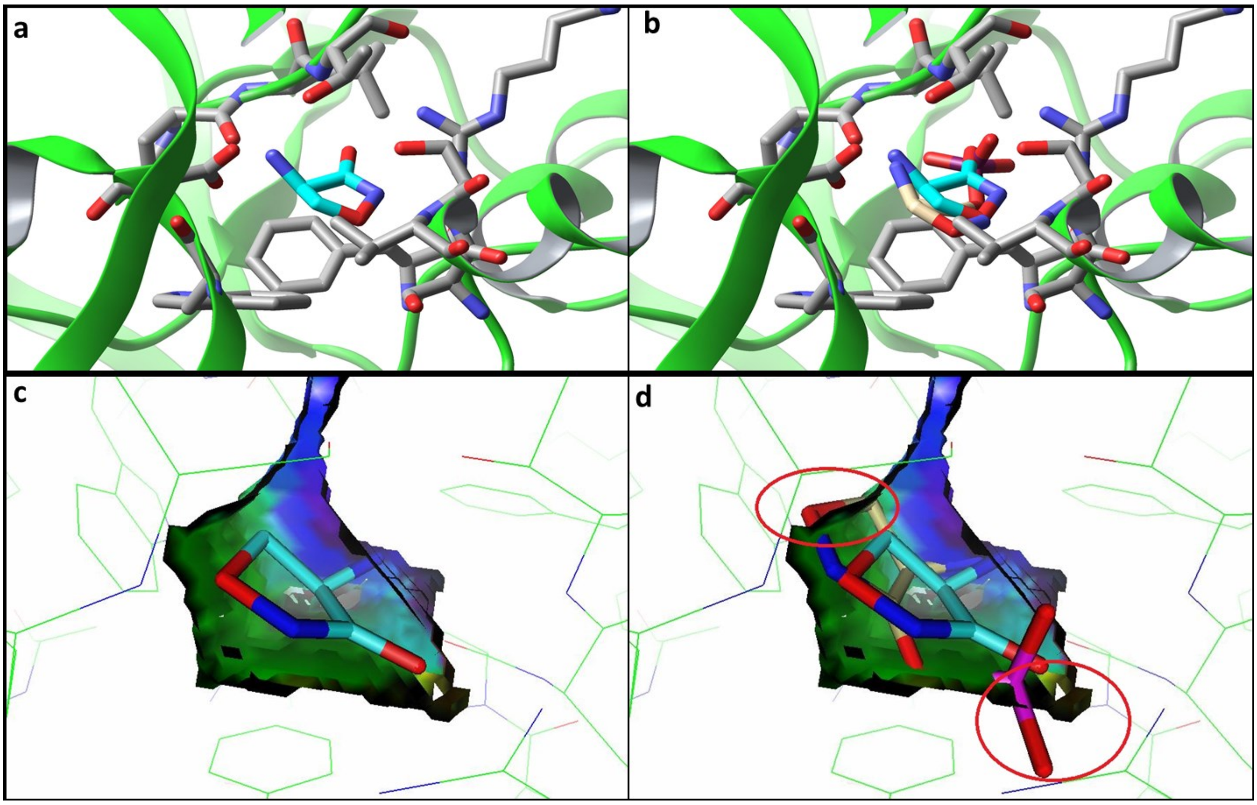

Supplementary Figure 6: Modelling of D-cycloserine and D-cycloserine-phosphate in the NMDA ligand biding site: (a) Crystal structure of the NMDA receptor with bound DCS (cyan) showing contacting residues; (b) NMDA structure with best-docked pose for DCSP (beige) also with location of DCS (cyan) overlayed for reference; (c) NMDA crystal structure showing location of bound D-cycloserine and surface of binding cavity (colour coded to indicate nature of surrounding residues; green = hydrophobic, red/blue = polar); (d) best-docked pose of DCSP (beige) within the cycloserine binding cavity of NMDA and overlayed with D-cycloserine (cyan) for comparison. The fit of DCSP is poor and the molecule violates the surface of the binding cavity in two places (red circles);

Supplementary References

1. Fan, C., Park, I.S., Walsh, C.T. & Knox, J.R. D-alanine:D-alanine ligase: phosphonate and phosphinate intermediates with wild type and the Y216F mutant. *Biochemistry* **36**, 2531-8 (1997).
